# Localization of the Elastic Proteins in the Flight Muscle of *Manduca sexta*

**DOI:** 10.3390/ijms21155504

**Published:** 2020-07-31

**Authors:** Henry Gong, Weikang Ma, Shaoshuai Chen, Geng Wang, Ramzi Khairallah, Thomas Irving

**Affiliations:** 1 Department of Biological Sciences, Illinois Institute of Technology, Chicago, IL 60616, USA; hgong7@hawk.iit.edu (H.G.); wma6@iit.edu (W.M.); schen89@hawk.iit.edu (S.C.); gengw@wustl.edu (G.W.); 2 Department of Cell and Molecular Physiology, Loyola University Chicago, Stritch School of Medicine, Maywood, IL 60153, USA; rkhairallah@myologic.com

**Keywords:** *Manduca sexta*, synchronous insect flight muscle, myofilament lengths, elastic proteins, sarcomere structures

## Abstract

The flight muscle of *Manduca sexta* (DLM_1_) is an emerging model system for biophysical studies of muscle contraction. Unlike the well-studied indirect flight muscle of *Lethocerus* and *Drosophila*, the DLM_1_ of *Manduca* is a synchronous muscle, as are the vertebrate cardiac and skeletal muscles. Very little has been published regarding the ultrastructure and protein composition of this muscle. Previous studies have demonstrated that DLM_1_ express two projectin isoform, two kettin isoforms, and two large Salimus (Sls) isoforms. Such large Sls isoforms have not been observed in the asynchronous flight muscles of *Lethocerus* and *Drosophila.* The spatial localization of these proteins was unknown. Here, immuno-localization was used to show that the N-termini of projectin and Salimus are inserted into the Z-band. Projectin spans across the I-band, and the C-terminus is attached to the thick filament in the A-band. The C-terminus of Sls was also located in the A-band. Using confocal microscopy and experimental force-length curves, thin filament lengths were estimated as ~1.5 µm and thick filament lengths were measured as ~2.5 µm. This structural information may help provide an interpretive framework for future studies using this muscle system.

## 1. Introduction

There are two major types of insect flight muscle: asynchronous and synchronous [1,2,3,4]. Asynchronous flight muscle is regulated by delayed stretch activation (SA), and contraction is uncoupled from neural stimulation [5]. This type of muscle is found in *Drosophila*, *Lethocerus*, *Bombus*, and many other species comprising more than two-thirds of the known flying insects [6,7,8,9]. The Mesothroacic Dorsolongitudinal Muscle (DLM_1_) in *M. sexta*, in contrast, is a synchronous flight muscle [10,11] where each contraction is coupled with a neural stimulation. This characteristic makes synchronous muscle less energy efficient so that insects with synchronous muscles, such as *M. sexta*, have substantially lower wingbeat frequencies compared to those with asynchronous muscle [12]. Tu and Daniel 2004 [13] proposed that the DLM_1_ can be used as a comparative model system to possibly elucidate aspects of mammalian cardiac muscle due to their similar physiological properties. In vivo, DLM_1_ muscle produces a narrow twitch length-tension curve and operates on the steep ascending limb, as does mammalian cardiac muscle [13]. Both cardiac and DLM_1_ muscle have large amplitudes of cyclical strain during contraction [13]. The DLM_1_, however, shows other intriguing physiological behaviors. It has been demonstrated that the dorsal regions of DLM_1_ are ~5.6 °C lower than the ventral regions [14]. This temperature gradient suggests that different regions have different functions, with warmer ventral muscle acting primarily as an actuator and cooler dorsal muscle acting primarily as a damper or spring when powering the downstroke of the wings [15]. Furthermore, the temperature gradient enables the storage of elastic energy in myofilaments and cross-bridges [16]. Whether such temperature dependent changes in function occur in other muscle systems is not known. Despite the promise of the DLM_1_ of *M. sexta* as an interesting model system for biophysical studies of muscle, not much is known concerning its structural, biochemical properties and how they determine the function of the DLM_1_.

Unlike birds, insects need to oscillate their wings at a higher frequency to fly due to their small body size [17]. Therefore, flight muscle requires adaptations in order to sustain this high frequency. Stiffness is a crucial physiological property in insect flight muscle (IFM) that enables rapid oscillatory contractions to power the wing strokes [15]. The overall stiffness of the muscle comes from the combined stiffnesses of the thick and thin filaments themselves, interactions between thick and thin filaments [16,18], and the elastic proteins with which they interact [19]. The elastic proteins in asynchronous IFM consist of projectin and kettin [20,21]. Projectin is a homolog to titin in vertebrate muscle. In vertebrate muscle, titin is an elongated protein that spans from the Z-band to the M-line. The PEVK domain and tandem Ig domain in the I-band region of titin can be stretched in response to stress [22,23]. In *Drosophila* and *Lethocerus* IFM, the N-terminal of projectin is attached to the Z-band, and the C-terminus is anchored to the thick filament in the A-band [20,21]. Projectin has a shorter PEVK region compared to titin, resulting in a relatively high stiffness in IFM fibers [20]. Kettin is a smaller polypeptide that is expressed, by alternative splicing, from the *sallimus* gene [24]. In *Drosophila* asynchronous muscles, this protein is found in the Z-band with its N-terminus attached to the thin filament and its C-terminus attached to the thick filament [25]. While these proteins have been well studied in asynchronous muscle, the characteristics of the elastic proteins in the DLM_1_, in particular their locations in the sarcomere, have not been established.

Previous work from our laboratory [26] showed that the DLM_1_ of *M. sexta* expresses two isoforms of projectin, two isoforms of kettin, and two large Sallimus (Sls) isoforms. Projectin, kettin, and Sls are all alternative splicing products of the *sallimus* gene [26]. The two projectin isoforms are 960 and 1050 kDa, respectively. The two kettin isoforms are ~500 and ~700 kDa, respectively. Both projectin and kettin isoforms in *M. sexta* are similar in sizes to *Lethocereus* and *Drosophila*. However, the two large Sls isoforms (~1.6 and ~1.2 MDa) had not previously been observed in *Drosophila* and *Lethocerus* flight muscles. The projectin isoforms in the DLM_1_ contain longer PEVK regions consistent with the observation that DLM_1_ can extend 8–10% [13] whereas the asynchronous IFM of *Lethocerus* can only stretch few a percent due to its shorter projectin isoform [20]. An open question was whether the orientations and positions of elastic proteins within the sarcomere of the DLM_1_ are the same as in asynchronous muscles, or is they show differences that could be related to their physiological differences. While the basic packing structure of the thick and thin filaments is similar in the DLM_1_ and other insect flight muscle, basic structural parameters, such as the length of the thick and thin filaments, have not previously been reported.

Here we used immuno-localization to determine the sarcomeric locations of the elastic proteins. By tagging the specific fragment of the proteins using antibodies, the results showed that the N-termini of both projectin and Sls are anchored to the Z-band of the sarcomere. Projectin spans across the I-band, and the C-terminus is in the A-band. Sls spans from the Z-band to the A-band, and the C-terminus is also in the A-band. By comparing confocal and immuno-labeled micrographs and length-tension curves, we were able to estimate the length of the thin filaments of the DLM_1_ as ~1.5 µm and the thick filament as ~2.5 µm, respectively.

## 2. Results

### 2.1. Filament Lengths

**Figure 1:**
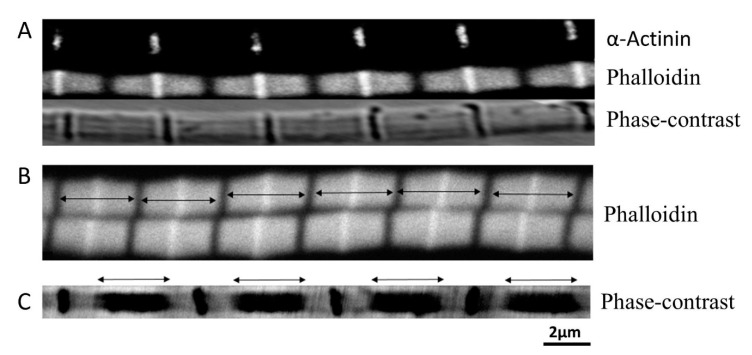
Estimating filament Length. (**A**) Top, Z-band labelled by α-actinin. Middle, actin labelled by phalloidin. Bottom, phase-contrast image of the fiber. (**B**) Phalloidin labelled thin filament length (double headed arrows) was defined by the distance between two adjacent H-zone (dark bands between two arrows). (**C**) Thick filament length (double headed arrows) was estimated after the fiber was mildly stretched.

Phalloidin specifically labels actin in the thin filaments. Phalloidin tagged with Alexa Fluor 633 was used here. The Alexa Fluor-phalloidin stained DLM_1_ myofibrils showed clearly labeled thin filament regions with well-defined edges, and with a brighter line is in the middle of each band (Figure 1A, middle). The brighter line in the middle of each band is most likely the Z-band. To verify that the bright line from the Alexa Fluor-phalloidin is the Z-band, the myofibril was tagged with an α-actinin antibody (MAC276). The result showed that α-actinin signals co-localized with the brighter signals of the phalloidin (Figure 1A top). This suggest that the brighter signal is from Alexa Fluor-phalloidin penetrating the Z-band, and the thin filaments from the adjacent sarcomere overlap with each other at the Z-band.

The myofibrils were mildly stretched (15% over slack length) while being observed under the microscope. Mild stretch can improve the Alexa Fluor-phalloidin signal by preventing neighboring actin filaments from the same sarcomere to overlap with each other (Figure 1A). By comparing the phase-contrast images with the Alexa Fluor-phalloidin labelled images (Figure 1A), the I-bands on either side of the Z-band were relatively short, even with 15% stretch from slack length. The intensity of the signal from fluorescently labeled anti- α-actinin antibodies across the Z-band was reduced to a one-dimensional projection. The peak intensity in the plot was fit to a Gaussian function to give the width of the peak. The width of the peak will reflect the thickness of the Z-band. The Z-band thickness measured this way was 0.14 ± 0.004 μm (*n* = 8) (Figure 1A).

The thin filament length was estimated by measuring the width of the fluorescent band (double-headed arrow in Figure 1B) from Alexa Fluor-phalloidin stained myofibril images. Each band represents two thin filaments plus a Z-band. The length of each band was measured at 3.18 ± 0.02 μm (*n* = 55). With a Z-band width of 0.14 μm, this would imply a thin filament length of 1.52 ± 0.01 μm. No noticeable I-band was seen in myofibrils at slack length, so it was impossible to measure thick filament length using phase-contrast images. However, the edges of the A-band (double-headed arrow in Figure 1C) were well defined in phase-contrast images from stretched myofibrils (100% over the slack length). Assuming these edges are defined by the length of the thick filaments, this yields an average length of the thick filaments of 2.5 ± 0.05 μm (*n*= 33).

### 2.2. Filament Integrity upon Sarcomere Extension

**Figure 2:**
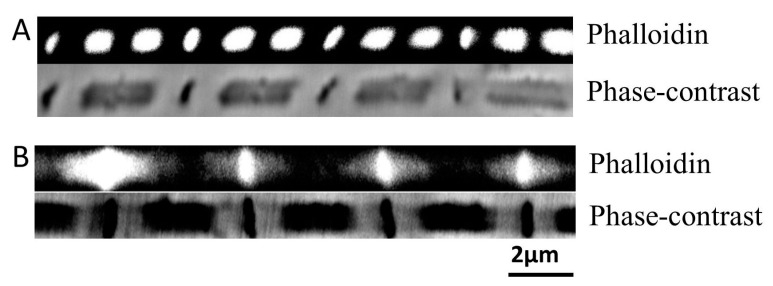
Filament integrity upon stretching. (**A**) Fiber was stretched manually to 5.4 μm. Top, actin labeled by Phalloidin. Bottom, phase-contrast image of the fiber. Phalloidin labeled actin was found concentrating in the A-band, leaving the I-band dark, indicating thin filaments have been detached from the Z-band. (**B**) Slow stretched fiber. Top, actin labeled by Phalloidin. Bottom, phase-contrast image. Slow stretch has left some phalloidin signal to be dispersed near Z-band, indicating some thin filaments have remained attached to the Z-bands.

When Alexa Fluor-phalloidin stained DLM_1_ myofibrils were stretched to ~5.4 μm from slack length in relaxing solution, the thin filaments appeared to be detached from the Z-band, and the Z-band remained intact (Figure 2A) as has been previously observed in *Drosophila* IFM [25]. We tested the notion that the thin filaments would remain intact when the myofibrils were stretched over longer time periods and with more gentle forces. Fibers were mounted on hooks and stretched slowly by about 100% (5%/second) with the motor, and then the fiber was fixed with PFM for phalloidin staining. In Figure 2B, the sarcomere lengths of this myofibril were about 6 μm, and most of the thin filaments were clearly pulled out of A-band. It cannot be excluded, however, that some broken actin filaments are left in the A-band under these conditions.

### 2.3. Force-Length Curve for M. Sexta Ventral Flight Muscle

**Figure 3:**
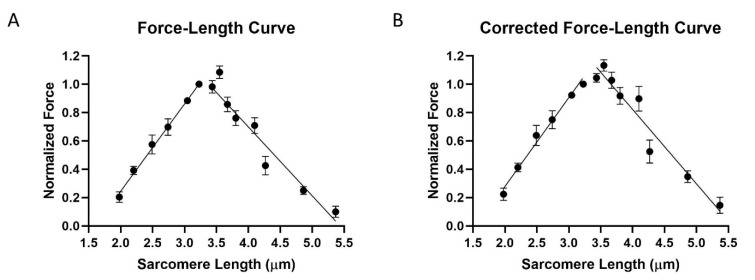
Force-length tension curve of outermost ventral muscle. (**A**) Length-force curve before correct for rundown. (**B**) Length-force curve after correction. Force is normalized to its value at 3.2 μm.

The classic length-tension experiments of Gordon et al., 1966, provide an alternative approach to investigate sarcomere architecture. In our experiments, chemically skinned DLM_1_ bundles in relaxing solution (see Methods) were stretched to a randomly chosen sarcomere length and then activated. The sarcomere lengths tested were 1.98 μm (*n* = 4), 2.2 μm (*n* = 4), 2.49 μm (*n* = 4), 2.74 μm (*n* = 11), 3.05 μm (*n* = 11), 3.23 μm, 3.44 μm (*n* = 4), 3.55 μm (*n* = 5), 3.67 μm (*n* = 10), 3.8 μm (*n* = 12), 4.1 μm (*n* = 6), 4.27 μm (*n* = 8), 4.86 μm (*n* = 9), and 5.37 μm (*n* = 9) (*n* stands for the number of fibers tested at each length). The maximum force in each trial was normalized against the force recorded at 3.23 μm. As shown in Figure 3A, the curve has an ascending limb from 1.98 μm to 3.23 μm, then a peak around 3.23 μm, and a descending limb from 3.45 μm to 5.37 μm. The data points plotted in the ascending limb, and the peak showed less than 25% rundown. That is, the force of the final activation was within 25% of the force from the initial activation, both measured at 3.23 μm. The muscle normally operates on the ascending limb of the force-length relationship [13], and it is difficult to stretch it to longer sarcomere lengths without damaging the sarcomere structure. Hence, the data will be more reliable on the ascending limb than on the descending limb. The ascending limb (1.98–3.23 μm) was fit using linear regression yielding an R^2^ value of 0.78, a slope of 0.62 ± 0.06 (S.E), and a x-intercept of 1.61 ± 0.04 (S.E). The descending limb (3.45–5.37 μm) was also fit with linear regression yielding an R^2^ value of 0.8, a slope of −0.49 ± 0.03 (S.E), and a x-intercept of 5.34 ± 0.14 (S.E).

The force-length curve was corrected by assuming that the rundown of each fiber was a linear function of the number of contractions (Figure 3B). The ascending limb was then fit using linear regression yielding an R^2^ value of 0.76, a slope of 0.63 ± 0.06 (S.E), and a x-intercept of 1.56 ± 0.07 (S.E). The descending limb was also fit using linear regression yielding an R^2^ value of 0.77, a slope of −0.53 ± 0.04 (S.E), and a x-intercept of 5.49 ± 0.15 (S.E).

### 2.4. Location of Projectin in the Sarcomere

**Figure 4:**
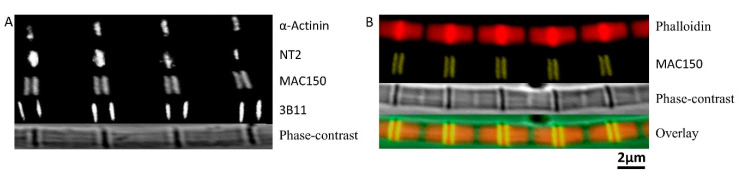
Location of projectin in the sarcomere. (**A**) From top to bottom. α-Actinin labeled Z-band, NT2 labeled N-terminal of projectin, MAC150 labeled middle portion of projectin, 3B11 labeled C-terminal of projectin, and phase-contrast image of the fiber. (**B**) From top to bottom. Phalloidin labeled actin, MAC150 labeled middle portion of projectin, phase-contrast image of the fiber, and overlaid image.

The orientation of projectin was determined by labeling DLM_1_ single myofibrils with antibodies raised against the N-termini, the C-termini, and the region in between both N- and C-termini of projectin. Antibodies used are described in Table 1 (see Methods). The NT2 antibody was used to label the N-terminal Ig9 and Ig10 peptides of projectin [27]. The results showed that NT2 signals was co-localized with α-actinin (Figure 4A) which indicate that, like kettin in *Drosophila* and *Lethocerus* flight muscle [28] and titin in vertebrate muscle [29,30], the N-terminus of projectin in DLM_1_ appears to be anchored to the Z-band.

The MAC150 antibody was used to label the fragments inbetween the N and the C-termini of projectin. While the location of the MAC150 epitope on projectin has not yet been precisely mapped, it has been used to identify projectin in *Lethocerus* [31] and *Drosophila* [32]. In mildly stretched myofibrils, the signals of MAC150 are located immediately next to the Z-band (Figure 4A,B), and overlap with the I-bands on either side of the Z-band (Figure 4B, yellow). To further demonstrate that MAC150 targets fragments of projectin in the I-band, we stretched the myofibril to a longer sarcomere length (3.9 μm). The results showed that at short sarcomere length (3.1 μm), the two stripes were close together, and the two stripes gradually move away from each other as the myofibril is stretched to longer sarcomere length. The position of the 3B11 epitope is located at the C-terminus of the projectin molecule [27]. While the 3B11 fluorescent signals were also observed next to the Z-band, they were further away from the Z-band compared to the MAC150 signals at slack length (Figure 4A).

### 2.5. Location of Sallimus (Sls) in the Sarcomere

**Figure 5:**
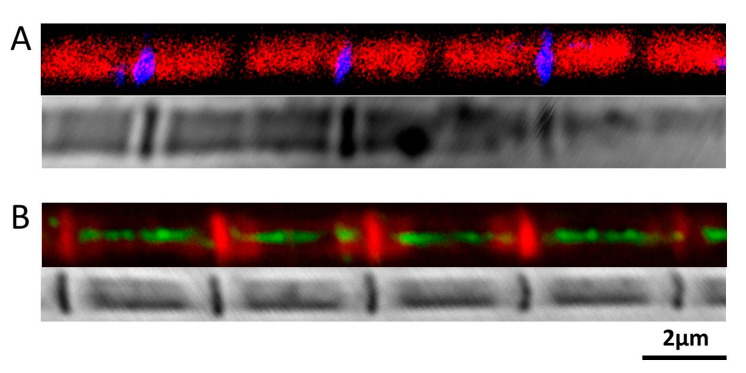
Position of Sallimus in the Sarcomere. (**A**) Top, N-Sls labelled N-terminal of Sallimus (blue) with phallodin labelled actin (red). Bottom, phase-contrast image of the fiber. The N-terminus of Sls is found in the Z-band. (**B**) Top, M-Sls labelled Sallimus (green) overlap with phalloidin labelled actin (red). Bottom, phase-contrast image of the fiber. M-Sls is found across the A and I-band.

The orientation and positions of Sls proteins were determined by labeling paraformaldehyde-fixed *M. sexta* IFM single myofibrils with custom made antibodies raised against peptides at the N-termini (between Ig1 and Ig2) and the middle part of Sls proteins (prior to the C-terminus, between Ig43 and Ig44). Myofibrils labeled with N-Sls antibodies raised against the N-terminal of Sls proteins showed that the fluorescent signals were co-localized with Z-bands (Figure 5A) as expected from the projectin experiment above. Both phalloidin staining and phase-contrast images confirmed that the N-Sls epitope was localized at the Z-band. The M-Sls antibodies stained in the A-band and I-band (Figure 5B). It clearly stained throughout the A-band except for the middle portion where the M-line is presumed to be located.

## 3. Discussion

### 3.1. Z-band Width in the DLM_1_

The Z-band width was determined by labeling it with fluorescently-labeled α-actinin antibodies (Figure 1A). The Z-band thickness was measured to be ~142 nm. In vertebrate muscle, the Z-band width is more or less well defined depending on fiber types. Usually, slow and cardiac muscle fibers have wide Z bands, and fast muscles have narrow Z-band widths [33]. The structures of insect flight muscle Z-bands are different from the structure of vertebrate muscle Z-band. Insect flight muscle Z-bands comprise the hexagonal lattice of the thin filaments produced by inter-digitation of thin filaments from adjacent sarcomere [34] connected by α-actinin. The Z-band thickness is 120nm in honeybee (*Apis* sp.) IFM [35], and honeybees fly with an average wingbeat frequency of 230 Hz [36] which is much higher than *M. sexta* wing beat frequency. By the same analogy from vertebrate muscle, it is reasonable to conclude that slower *M. sexta* IFM has a wider Z-band thickness than does honeybee IFM. It should be noted, however, that the measured width from the fluorescence confocal image will be the convolution of the actual width with the resolution function of the confocal microscope., about 140 nm under our conditions. This implies that the width of the Z-band could be smaller than our estimate of 142 nm so that 142 nm represents an upper bound. However, since the thickness of the Z-band agrees with other observations made by electron microscopy in other model systems it is unlikely to be significantly smaller (under 100 nm).

### 3.2. Breakage of Thin Filaments with Stretch under Relaxing Conditions

It may be surprising to see broken thin filaments when muscles are stretched under relaxing conditions but these have been observed in other insect flight muscle systems including *Drosophila* [25]. Two possible explanations can be proposed for this observation. First, even though the muscles were in relaxing solution, there are still some kind of actin-myosin interactions remaining. One possibility interaction could be the so-called troponin bridges linking the thick filament and troponin on the thin filaments that have been observed in relaxed *Lethocerus* indirect flight muscle [37]. A second possibility is that elastic proteins present in the sarcomeres link actin and myosin filaments hindering the removal of actin filaments from the A-band. Supporting this notion is the observation that actin filaments could be pulled out of the A-band more easily after upon digestion of kettin by μ-calpain in *Drosophila* IFM myofibrils [25] All of these possible linkages are unlikely to be permanent since pulling the actin filaments out slowly mitigated this behavior.

### 3.3. Force-Length Curves

Contraction in striated muscle requires the binding of myosin heads on the thick filament binding to specific sites on the thin filament to form cross-bridges. The change in force is dependent on the number of cross-bridges formed between the thick and the thin filaments. The maximum force that a muscle fiber can generate is when all myosin heads along the thick filament are interacting with the neighboring thin filament. When the myosin heads are prevented from binding to the thin filaments, the muscle fiber produces no force [38,39,40].

The classic work of Gordon et al., 1966, provides an interpretative framework for our findings. When viewed by light microscopy (see, e.g., Figure 1), the sarcomeres in the insect flight muscle show a similar longitudinal alternating A-band, I-band structure as in vertebrate striated muscle, and the DLM_1_ length-tension curve has a similar shape to that seen from cardiac muscle [13]. Thus, we can apply the analysis used in Gordon et al., 1966 to estimate the lengths of the myofilaments in the DLM_1_. Gordon et al., 1966 demonstrated that the length-tension curve from intact frog muscle could be separated into four regions, going from short to long sarcomere lengths, first a steep slope ascending region, then a moderate slope ascending region, a plateau region, and a descending region. Based on the sliding filament theory, these four regions correspond to different degrees of overlap between thin and thick filaments. In the steep ascending region, the Z-bands are closer to each other so that they press against the ends of the thick filaments and the thin filaments overlap with neighboring thin filaments from the other end of the sarcomere. This results in fewer binding sites on the thin filaments available for the myosin heads to bind. As sarcomere length increases, the Z-bands gradually move apart, decreasing the amount of overlap between thin filaments making more binding sites available for myosin heads to bind (moderate ascending region). In the plateau region, the thin-thin filament overlaps are abolished so that all binding sites along the thin filaments are available for myosin heads to bind, and the interactions of thick and thin filaments are maximized. The length of the plateau region is determined by the length of the so-called bare zone in the center of the thick filaments, which lack myosin heads, so the force is constant as thin filaments transverse this region. As sarcomere length continues to increase (descending region), the Z-bands move further apart, pulling the thin filaments out of the A-band, so there are fewer actin-binding sites for the myosin heads. Thus, the force decreases linearly towards zero in the descending region.

The force-length curves in Figure 3 from skinned DLM_1_ are consistent with the sliding filament theory as described above. Moreover, they are broadly similar to the that of membrane intact DLM_1_ shown by Tu and Daniel et al., 2004 [13], both being relatively narrow and steep curves as observed in mammalian cardiac muscle. The data points in Figure 3A for the ascending limb consist of data points with less than 25% rundown. However, we did not obtain any data points on the descending limb that had less than 25% rundown. A possible explanation is that stretching and activating at long sarcomere lengths inflicted irreversible damage to the sarcomere structure. Based on previously published data, intact DLM is only capable of extending 8% to 10% from rest length [13]. Elastic proteins, such as projectin, in the DLM_1_ are shorter compared to those in vertebrate muscle [26]. Furthermore, our immuno-labelling experiment demonstrated that rapidly stretching the fiber from slack length can result in detachment of thin filaments from the Z-band. We took care to stretch the fibers gently, but we cannot exclude the possibility that some thin filaments are detached at long lengths in our preparation (see results above). Therefore, the descending limb of the force-length curve should be considered less reliable compared to the ascending limb.

Knowing that the descending limb possibly contains points from damaged fibers, the steep slope shown in Figure 3A could be from the damaged fibers generating less force than if the fiber were undamaged. In other words, if the sarcomere structure remained intact at long sarcomere lengths, the slope of descending limb should be less steep than what have shown here, and the thin-thick filament overlap would be abolished at a sarcomere length that is longer than 5.34 μm (Figure 3A). To test this hypothesis, we applied a correction to the data values assuming that the rundown is a linear function of the number of contractions. The corrected curve (Figure 3B) retains the same shape as the uncorrected curve but is less steep and less narrow (Figure 3A). The sarcomere length that abolished thin-thick filament overlap also increased to 5.49 μm. We speculate that 5.49 μm is closer to the true value for the zero-force intercept of the extrapolated line for the descending limb if the fibers remained intact.

### 3.4. Thick and Thin Filament Lengths

Based on the Gordon et al., 1966, interpretation, we can estimate both thin and thick filament lengths for the DLM_1_ using our results. We analyzed our data using two methods (Figure 3, see results). In both curves, 3.23 μm, the force reached its maximum, indicating that the thin-thin filament overlap has just been eliminated. So that thin filaments from each end of the sarcomere are aligned tip to tip in the H-zone. This implies that the length from one Z-band to another is ~3.23 μm. The width of a Z-band cannot be estimated from Figure 3. But by subtracting the width of the Z-band from immuno-localization of 0.14 μm, the length thin filament is estimated to be 1.52 μm, consistent with the estimate from the immuno-labeling experiment of 1.52 ± 0.01 μm. As discussed above, because of the limited resolution of the microscope, the Z-band width may be smaller than 0.14 μm but the estimates for filament length obtained by the two methods will still be the same within experimental error.

The length of the thick filament can also be estimated by using the data shown in Figure 3. Starting at 3.44 μm, as sarcomere length increases, the thin filaments are being gradually pulled out of the A-band, resulting in a linear decrease in the amount of overlap with the thick filaments [40]. As a result, force also decreases linearly [40]. We assume that one thin filament is aligned tip to tip with its neighboring thin filament at a sarcomere length of 3.23 μm, and thin-thick filament interactions were abolished when the force equals zero. Based on our regression line of the descending limb, thick-thin filament overlap is abolished at 5.34 ± 0.14 μm for Figure 3A, and 5.49 ± 0.15 μm for Figure 3B. Then the length of the thick filament is estimated to be 2.11 ± 0.23 μm (S.E) and 2.26 ± 0.32 μm (S.E) from Figure 3A,B, respectively. Both length values calculated using this method are lower than the value estimated from the immuno-localization experiment (2.5 ± 0.05 μm) but the larger estimate (2.26 ± 0.32 μm) is within experimental error. As described above, the substantial amount of rundown in fibers stretched into the descending limb of the force-length curve suggests that the fibers were damaged, probably due to breakage of the thin filaments, so that the slope with undamaged fibers would be less steep than what is shown in Figure 3. Our correction for rundown assumes a linear process that may or may not be the case, but we speculate that the thick filament length estimated using the data in Figure 3B is closer to true length of the thick filament than the value from Figure 3A. In any event, considering the large experimental error in the mechanical data and the relatively small error in the microscopic data, we estimate the length of thick filament to be about 2.5 μm.

Our results showed that force reaches its maximum around 3.23 μm, then started to decrease at around 3.45–3.55 μm. Given the scatter in our data, this result suggests that DLM_1_ has, at most, a small plateau region, as also suggested by Tu and Daniel et al., 2004 [13]. This implies that thick filaments in the DLM_1_ might have a small or non-existent bare zone. This question could be best resolved by future electron microscopy studies.

### 3.5. Immuno-Localization of Projectin

The orientation and location of projectin in the sarcomere was determined by immuno-localization. The N-terminus of projectin is inserted inside the Z-band (Figure 4). Previous studies in *Drosophila* and honeybee indicated that projectin extends from the Z-band toward the A-band to just overlap the tip of the myosin filaments [35,41]. The 3B9 anti-projectin immuno-electron microscopy staining of beetle flight muscle at rest showed that the stripes were approximately 180 nm inside from the edge of A-band [42]. Given the distance from the 3B11 epitope to the Z-band was about 380 nm and the relatively short I-band width, it is reasonable to propose that the end of projectin is attached somewhere inside the edge of the A-band, not just linking the tips of the myosin filaments. Therefore, the projectin molecule is oriented with its N-terminus embedded in the Z-band, and the C-terminus somewhere inside the A-band. Comparing the MAC150 signals to the phase-contrast myofibril images, the MAC150 signals are localized in the I-band. A single projectin molecule is approximately 300 nm long in crayfish claw muscle [43,44]. Asynchronous insect IFM has a range of I-band lengths ranging from 0.1 μm to 0.2 μm [45]. *M. sexta* flight muscle has longer sarcomere lengths with longer I-bands than asynchronous *Lethocerus* flight muscle. The MAC150 epitope, therefore, is most likely located in the I-band, where the extensible regions are located. This result also suggests that the MAC150 epitope is somewhere in the middle of the projectin molecule.

### 3.6. Immuno-Localization of Sls Proteins

Like all the elastic proteins, titin in vertebrate muscle, and projectin and kettin in insect muscle [28], the N-termini of Sls proteins of the DLM_1_ are also inserted into the Z-band (Figure 5). The M-Sls antibody labeled the entire A-band except at the location of the M-lines. The M-Sls antibody was monoclonal and it is presumed to recognize only one epitope. There are probably multiple homologous domains existing along the entire length of the Sls protein so that the fluorescent signals come from the entire A-band. The same phenomenon has been seen in other cases. The 3B9 antibody, a monoclonal projectin antibody stained both sides of the Z-band in beetle flight muscle, stained the entire A-band, except for the middle portion in beetle leg muscle [46]. SM1, a monoclonal antibody to vertebrate striated muscle connectin (another name for titin) stained both the I- and A-band, except for the location of the Z-band and the M-line [35]. The widths of M-Sls antibodies labeled bands were much smaller than the width of the myofibril in both phalloidin and phase contrast images (Figure 5B). In contrast, the N-Sls antibody stained the entire Z-band (Figure 5A). While M-Sls will only recognize Sls, N-Sls also recognizes kettin, and both kettin and Sls have their N-termini anchored in the Z-band [25]. In vertebrate muscle, there are on average 6–12 titin molecules per thick filament, and we can assume that the molecular weight of each titin molecule is 3 MDa [47]. We can also assume the length of thick filament to be 1.6 μm and the molecular weight of each thick filament is about 55MDa, so that the mass ratio of myosin to titin is estimated from 1:1.5 to 1:3. However, based on our findings, the mass ratio of myosin over Sls proteins is ~10 in *M. sexta* flight muscle. This implies that there is relatively much less Sls protein present in *M. sexta* flight myofibrils than there is titin in vertebrate muscle, less than one Sls molecule/thick filament on average. Given that the immuno-fluorescent bands are narrower than the myofibril width, this suggests that high molecular weight Sls proteins (> 1 MDa) are only found in the center of the myofibrils, as has been previously reported for the smaller Sls (700) isoform found in *Drosophila* IFM [24].

## 4. Materials and Methods

### 4.1. Muscle Dissection for Immuno-Localization Experiments

**Figure 6:**
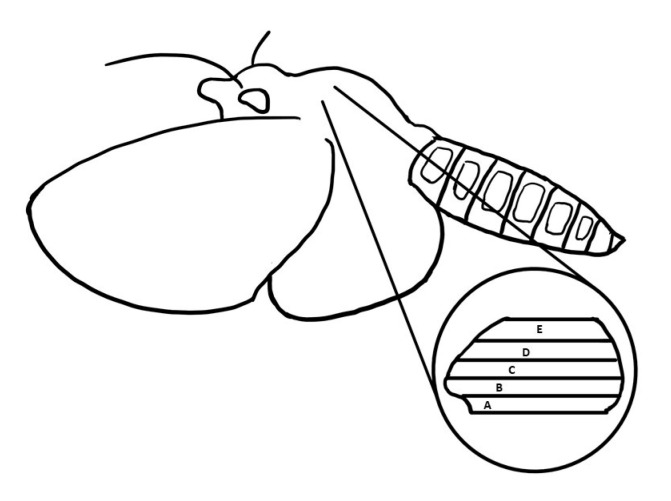
A schematic representation of a *Manduca sexta* moth and the location of the DLM_1_ flight muscle. The five layers of muscle are labeled from A to E with A being outermost ventral muscle and E being the outermost dorsal muscle.

Moths (*M. sexta*) were shipped overnight from the Department of Biology at the University of Washington (courtesy of Dr. Tom Daniel). The head, wings, abdomen, and legs were first removed from the body using scissors. All the hairs in the thorax were removed using by wetted Kimwipes^TM^ tissues. The thorax encompassing the DLM_1_ was sliced into two segments at the median sagittal plane using a razor blade and placed in skinning wash solution (100 mM KCl, 10 mM MOPS, 5 mM EGTA, 9 mM MgCl_2_, 4 mM Na_2_ATP, 20 mM 2,3-Butanedione monoxime (BDM), and one tablet of Protease inhibitor cocktail (PIC), pH 6.8) for dissection. The DLM_1_ consists of five subunits (Figure 6). The dorsal part of the DLM_1_ was obtained from subunits D and E, and the ventral part of the muscle was dissected from A and B subunits. Different subunits were separated with a blunt probe and scissors. Isolated muscle fibers were soaked in skinning solution (skinning wash with 1% TritonX-100) at 4 °C for 8 to 12 h. After skinning, the muscle fibers were washed with skinning wash solution three times, for 10 min each time.

### 4.2. Fiber Preparation for Immuno-Localization

Skinned muscle fibers were divided into three groups: free fibers, unstretched fibers, and stretched fibers. Each fiber bundle was attached to T-clips at both ends in PBS filled Sylgard^TM^ silicon elastomer plate. The stretched fibers were stretched as far as possible and pinned down using insect pins. The unstretched fibers were simply pinned down with insect pins. Then the fibers were incubated with 2% paraformaldehyde (PFM) in phosphate-buffered saline (PBS) for 10 min for fixation. After 10 min, the fibers were washed with PBS solution twice, for 5 min each. The sarcomere integrity was checked under a 40× light microscope. Only fibers with clear striation patterns were used for immuno-localization experiments.

### 4.3. Myofibril Preparation for Immuno-Localization

Skinned fibers were divided into two groups: free end and stretched fibers. The protocol for stretched fibers was as described above. Muscle fibers were fixed with 2% PFM for 10 min. In total, 2–3 pieces of muscle bundles (no more than 0.05 g) were incubated in myofibril isolation solution (20 mM MOPS, 5 mM MgCl_2_, 5 mM EGTA, 5 mM ATP, 5 mM DTT and PIC (Roche) solution 1:100 dilution, pH. 7) with 50% glycerol and 0.5% TritonX-100 into a flat end tube on ice for 2 h to dissolve all the extracellular debris. The muscle fibers were homogenized for 30 s on ice using a Tissue Tearor^TM^ homogenizer. The suspension was transferred into centrifuge tube, and the tube was centrifuged at 4 °C, 10,000× *g* (8750 rpm, Eppendorf model 5415c) for 10 min, and then the supernatant was discarded. The pellet was re-suspended in 1mL myofibril isolation solution with 50% glycerol. The myofibril preparation was checked under a light microscope with a 40× objective. Only myofibrils that were clean and well separated were used for further experimentation. The wash cycle was repeated more times if necessary. The myofibrils were then ready for further experimentation. PFM fixed myofibrils were stored in PBS for one week at 4 °C.

### 4.4. Antibody Labeling of Myofibrils

Three ~1 × 1 cm squares were drawn with a hydrophobic pen on a glass microscope slide. About 250μL blocking solution (8% bovine serum albumin (BSA) and PBS) was added to each square. Permeated and fixed fiber bundles were then peeled into very thin fibers, as thin as possible, then the fibers were put into blocking solution and incubated overnight at 4 °C. The blocking solution was then carefully removed with a pipette without disturbing the fibers. Fibers were incubated with primary antibody (Table 1) in antibody incubation solution (3% BSA-PBS) for 1 h at room temperature. After an hour, fibers were rinsed carefully with antibody incubation solution twice for 5 min each time. Then the fibers were incubated in secondary antibody solution (Table 2) with phalloidin for 1 h at room temperature avoiding exposure to light (5 uL phalloidin in 200 uL solution). The fibers were rinsed carefully with PBS twice, 5 min each time. The solution was aspirated as much as possible, using paper tissue if necessary, to remove any remainder. A half drop of prolong gold anti-fade (Invitrogen, Carlsbad, CA, USA) was put onto the fibers and covered with a long coverslip (50 mm). Well-separated single myofibrils were also labelled with antibodies using this procedure. Exposure to light was avoided at all times after incubating with the secondary antibodies. All antibody labelled samples were observed under a Leica TCS confocal microscope (Leica Microsystems, Buffalo Grove, IL, USA) with a 100× oil-immersion lens with a CCD camera with pixel dimensions of 1024 × 1024. The Leica TCS SP2 software (Leica Microsystems, Buffalo Grove, IL, USA) was used to acquire the images.

### 4.5. Force Measurement and Data Recording for Passive Stretch Experiments

The skinned muscle fiber bundles were washed with skinning wash solution three times, for 10 min each, prior to usage. The fiber bundles were attached to two hooks with glue made from cellulose nitrate dissolved in acetone. One hook was attached to a dual-mode force/length servo-motor (300C, Aurora Scientific, Aurora, ON, Canada) attached to a X-Y-Z positioner. The other hook, the mounting hook, was attached to another X-Y-Z positioner, and it was held stationary during the experiment. The data acquisition system (National Instruments cDAQ 8178, Austin, TX, USA) was connected to the data acquisition computer via a USB cable. The force and length output as a function of time were recorded by a custom Labview (v.8.5.1, National Instruments) computer program.

Once the glue was dry, the fiber was lowered into a chamber filled with relaxing solution (91 mM K-pro, 3.5 mM MgCl_2_, 2.2 mM Na_2_ATP, 7 mM EGTA, 20 mM Imidazole, 15 mM Na_2_CP, 1.49 mM BDM, 3 units CPK, and 1 tablet of PIC (Roche, Mannheim, Germany), pH 7). The fiber length was adjusted by slowing adjusting the X-Y-Z positioner until the passive force was about to generate. The final position of the two hooks were recorded, and this was taken as the slack length of the muscle. Sarcomere length was measured using a He-Ne laser.

### 4.6. Muscle Preparation for Force-Length Curves

The thorax of the moth was dissected as describe above. The outermost ventral muscle (Figure 6, Region A) was isolated in relaxing solution under a dissecting microscope. BDM has been demonstrated to protect the muscle during dissection. The ventral muscles were further separated into small bundles and pinned down to a Sylgard^TM^ substrate in a petri dish containing skinning solution (relaxing solution and 1% TritonX-100). Then the preparation was placed on top of a shaker at 4 °C for 1 h. After skinning for 1 h, the muscle fibers were washed with Trehalose solution (600 mM Trehalose in relaxing solution, pH 7) three times for 5 min each on the shaker to remove the Triton. Then, the muscle fibers were transferred to storage solution (75% Trehalose solution and 25% glycerol) at 4 °C for 15 min before placed in −80 °C for long-term storage for a maximum of three to four weeks.

### 4.7. Force Measurement and Data Recording for Force-Length Curves

The fibers were removed from the −80 °C freezer and washed with the relaxing solution three times, for a total of 15 min, to allow the glycerol to diffuse out of the fiber. The fiber bundle was further split into smaller bundles (0.089 ± 0.002 mm (S.E)) in fresh relaxing solution under a dissecting microscope. Each end of the muscle bundles was glued (with nitrocellulose dissolved in acetone) to a stainless steel post, one connected to a force transducer (Aurora Scientific Inc., model 402A, Aurora, ON, Canada) that is attached to an X-Y-Z positioner and the other one is a fixed post attached to a separate X-Y-Z positioner. The force transducer was connected to an ASI 610A data acquisition and control system (Aurora Scientific Inc.) to record force. The data was recorded at 200Hz. The sarcomere length was adjusted to the desired length using light diffraction with a He-Ne laser.

Once the glue on the posts were dried, the fiber was lowered into a custom designed chamber containing 1 mL of relaxing solution. When the force has stabilized, the data collection was initiated. The activation solution (90 mM K-pro, 3 mM MgCl_2_, 7.1 mM CaCl_2_, 2.3 mM Na_2_ATP, 7 mM EGTA, 20 mM Imidazole, 15 mM Na_2_CP, pH. 7) was manually exchanged with the relaxing solution in the chamber after few seconds of waiting. When the force has reached a plateau, the solution was exchanged with relaxing solution and data collection was terminated. Each preparation was activated three to five times. Rundown is calculated as the difference in force between the first activation and the last activation at a sarcomere length of 3.23 μm. The active force was calculated as the difference between the plateau region of the contraction and the baseline force under the relaxing condition using DMAv5.300 software from Aurora Scientific Inc. The active force at various lengths were normalized to the force collected at sarcomere length 3.23 µm. The width of the fiber bundle was measured at a sarcomere length of 3.23 µm under a stereo microscope at 5× magnification.

## 5. Conclusions

This study provides characterization of the location and orientation of the elastic proteins of the flight muscle DLM_1_ in *Manduca sexta.* The results showed that the DLM_1_ has a similar Z-band width compared to some asynchronous flight muscles. Projectin extends from the Z-band to the A-band of the sarcomere. The Sls proteins initiate in the Z-band and terminate in the A-band, close to the M-line. The length of the thick filaments and the thin filaments are estimated as ~2.5 μm and ~1.5 μm, respectively. This information will help in interpreting future biophysical studies of this interesting synchronous muscle model system.

## Figures and Tables

**Table 1:** Primary antibodies for immuno-localization. Source A: Agnes Ayme-Southgate, College of Charleston; Source B: Babraham Biosccience Technologies, Cambridge University UK; Source C: Custom made from GL Biochem (Shanghai, China) Ltd.

Clone	Protein	Host	Dilution	Antibody	Source
MAC150	Projectin	Rat	1:1000	Monoclonal IgG	B
MAC276	α-Actinin	Mouse	1:300	Hybridoma IgM	B
NT2	Projectin	Rabbit	1:200	Polyclonal	A
3B11	Projectin	Mouse	1:200	Monoclonal	A
N-Sls	Sallimus	Mouse	1:100	Monoclonal	C
M-Sls	Sallimus	Mouse	1:100	Monoclonal	C

**Table 2:** Secondary Antibodies for Immuno-localization.

Name	Fluor	Host	Dilution	Antibody	Source
Anti-Rat	Cy3	Goat	1:1000	Polyclonal	ThermoFisher
Anti-Mouse	Cy5	Goat	1:1000	Polyclonal	ThermoFisher
Anti-Rabbit	A488	Goat	1:1000	Polyclonal	Abcam

## Abbreviations

ATP	Adenosine triphosphate
BDM	2,3-Butanedione monoxime
BSA	Bovine serum albumin
CaCl_2_	Calcium chloride
CPK	Creatine phosphate kinase
DLM_1_	Mesothoracic dorsolongitudinal muscle
DTT	Dithiothreitol
EGTA	Ethylene glycol-bis(β-aminoethyl ether)-N,N,N′,N′-tetraacetic acid
HCl	Hydrochloric acid
IFM	Insect flight muscle
K-Pro	Potasium propionate
MgCl_2_	Magnesium chloride
MOPS	3- (N-morpholino)propanesulfonic acid
Na_2_ATP	Adenosin 5′-triphosphate disodium
Na_2_CP	Creatine phosphate disodium
NaOH	Sodium hydroxide
PBS	Phosphate-buffered saline
PFM	Paraformaldehyde
PIC	Protease inhibitor cocktail
Sls	Sallimus protein
